# Using the LCA Method to Develop the Production of Pigment for Processing Plastics

**DOI:** 10.3390/ma16165524

**Published:** 2023-08-08

**Authors:** Patrycja Bałdowska-Witos, Andrzej Tomporowski, Marek Bieliński

**Affiliations:** Faculty of Mechanical Engineering, Bydgoszcz University of Sciences and Technology, Al. Prof. S. Kaliskiego 7, 85-796 Bydgoszcz, Poland; a.tomporowski@pbs.edu.pl (A.T.); biel@pbs.edu.pl (M.B.)

**Keywords:** polymers, PET, pigments, environment, energy consumption

## Abstract

In recent years, the chemical industry has been developing more and more dynamically, which results in the introduction of many new chemical substances to the market. However, some of them do not meet the accepted standards and may be toxic to humans and the environment. This problem largely concerns polymer materials, which are currently widely used in many areas of the economy. This is indirectly related to the coloring of these materials during processing. Therefore, it became necessary to introduce modern research procedures that enable the quantitative and qualitative determination of the impact of coloring agents in the processing of plastics, in order to include their negative impact on humans and the natural environment. The LCA methodology was used in this work, with ReCiPe 2016 used as the test method. Among the analyzed technological operations, the highest negative impact on the environment was characterized by the process related to heating the tested material (2.08 × 10^−1^ Pt). Among the materials, polyethylene terephthalate was distinguished by the greatest harmful effect on human health (2.91 × 10^−1^ Pt) and the quality (2.35 × 10^−2^ Pt) of the environment. The use of recycling processes would reduce the negative impact on human health (about −3.71 Pt), the ecosystem (about −0.14 Pt), and resources (about −0.27 Pt).

## 1. Introduction

**Figure 1:**
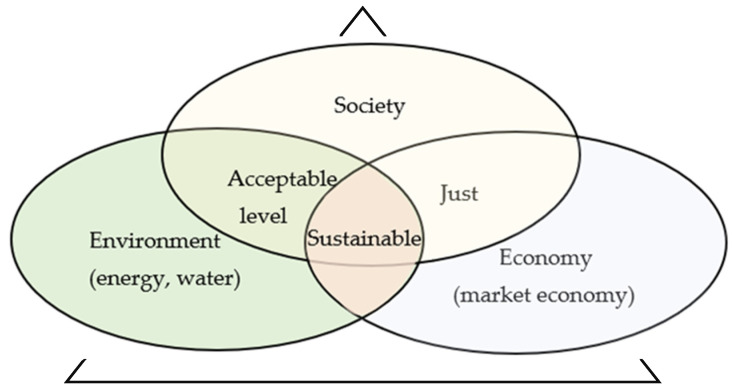
Multidimensional aspects of sustainable development.

Sustainable development is defined as socio–economic development, in which, there is an integration of economic and social activities while maintaining human rights, natural balance, and the sustainability of basic natural and environmental processes [1,2]. According to the definition formulated almost three decades ago [3], development understood in this way is aimed at ensuring “the fulfilment of the needs of the present generation without compromising the possibility of satisfying the needs of the next generations”. Thus, the sustainable development of producing colorants for processing plastics can also be treated as a process, whereby three basic aspects are taken into account: economic, ecological, and social (Figure 1).

The industry specificity of polymers may constitute a source of threats, which varies in terms of nuisance, for individual elements of the natural environment: soil, water, air, plants, animals, and humans. This mainly concerns the production of elements with a short service life (packaging) and, at the same time, elements with a lifetime of 20–50 years (water and gas installations, PVC window profiles, etc.). Several publications have described in detail the problems associated with energy efficiency and the environmental impact of injection technology [1,4,5]. Moreover, in the research, references were made to selected techniques for manufacturing injection molds, the injection process itself, and injection-molding machines, as well as proposing indicators specific to this technology.

The analysis, evaluation, development of processing plastics, and environment include water and sewage management, the use of energy carriers, the management of by-products, waste management—with particular emphasis on post-consumer polymer waste—protection of air against pollution, especially dust and smell, soil protection, and noise reduction.

**Figure 2:**
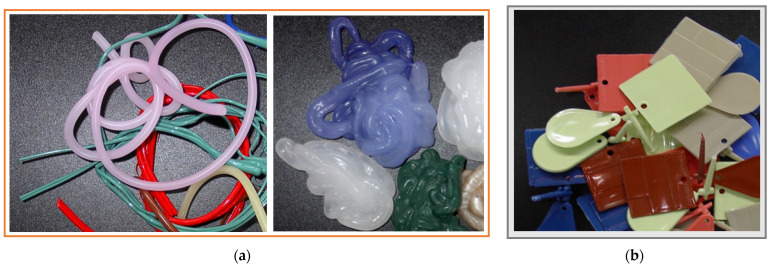
Various forms of technological wastes from the production of color concentrates: (**a**) extrusion; (**b**) injecting (own study).

The basic task in the field of analysis, evaluation, development of processing plastics, and the environment is to reduce water intake and reduce the amount of wastewater, the emission of pollutants into the air, and waste generation. However, these outcomes remain possible by controlling the individual production processes and analyzing the possibilities of introducing necessary improvements. This applies to both producing finished products, as well as intermediates used in the subsequent processing steps (e.g., the production of modifiers, including pigments). Figure 2 presents photographs of various forms of technological waste, generated during the production of masterbatches.

New technological solutions and innovations may reduce the environmental impact of production installations or the entire plant. Guides on the application of the best available production techniques, which are not obligatory, although provide knowledge, can be of significant help since they take into account the latest results from scientific research and various solutions that have been verified in similar plants in the specific industry.

**Figure 3:**
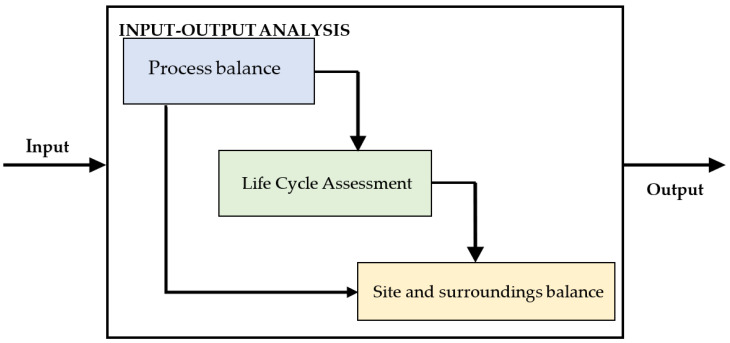
An example of the ecological balance sheet structure in an enterprise (own study).

Determining the scope of the negative consequences production plants cause on the natural environment is possible by conducting a constant analysis of the various stages of production processes and operations as well as the existing infrastructure. Moreover, it can be used to determine the needs in the field of environmental impact and indicate the possibilities of reducing or completely eliminating this impact, while assessing the effects of the implementation of pro-ecological projects. The task of sustainable development for plastic processing plants includes quantitative and qualitative analysis of processed raw materials and finished products, as well as monitoring the consumption of energy carriers, wastewater production, and waste and gas emissions into the atmosphere. One of the tools enabling this analysis, assessment, processing development, and identification of environmental problems is ecological balance, which is an example of an analysis aimed at the comprehensive identification of factors influencing the environment by enterprises [6,7,8]. The input and output balance sheets (Figure 3) include an inventory of all incoming and outgoing streams of materials, energy carriers, pollutant emissions, and primary and by-products.

Implementation of the ecological balance enables the identification of the aforementioned areas in the production process or individual operations, whereby material consumption may be reduced, energy efficiency increased, or pollution emissions reduced. In this context, it should be noted that some of the authors [9,10] paid attention to the determinants of eco-efficiency, the introduction of eco-innovations, and the implementation of cleaner production (Table 1).

This paper proposes a new method of assessing the pigment production process, which is to reduce the effects on the environmental impact. The main purpose of this study was to search for interactions between the elements that reduce energy consumption in the production process and the effective use of low-waste technologies. Among the objectives of the work was an attempt to synthesize the available literature on sustainable energy and environmental management in polymer plastics.

It was recognized that the main goal of the work could be achieved by solving two problems: what criteria should be adopted to conduct a creative analysis and evaluation of product quality, process efficiency, and harmlessness of the product and process?

## 2. Models, Materials and Methods

### 2.1. Energy Consumption in Production and Consumption of Materials

An important direction in the development of all production plants is the creation of indcators that reduce the energy consumption in production, while simultaneously increasing energy efficiency. Within the selection of indicators, it was considered the most important: indicators that enable the analysis, evaluation, and, above all, environmentally sustainable development in the production process of plastic pigments.

To facilitate and refine the data collection, special inventory tables were created. Data collection allows for the precise determination of the source of origin; here the geographical scope for the production technology was located in central Poland [11]. The data comes from a production plant, which has operated in the country since 2021. Table 2 and Table 3 contain the collected results of the research on the consumption of energy carriers and materials, as well as examples of residues, wastes, and by-products in the production of coloring materials for polymer materials. The entire process is diversified in terms of the unit consumption of auxiliary materials, which justifies the need to search for and implement cleaner production methods [12] by managing the generated waste and post-production residues.

In the processing of plastics, the aim is to use closed circuits to cool the water and waste heat to heat spaces and utility water [13]. The greatest amount of water is used to cool materials during the production process. Using the cold granulation method in the production of pigments is related to the need to cool the plasticized polymer material to a state that allows its fragmentation.

### 2.2. Description of the Research Object

Masterbatch is coloring concentrates in the form of granules and is produced using cold and hot technologies. They are used to pigment or modify polymer properties. The proportion of pigments and modifying agents in the concentrate is higher than in the final product. The concentrates are precisely dosed (volumetrically or gravimetrically) and mixed with the polymer raw material during the processing.

Compared to other agents, such as pastes, powder mixes, or liquid pigments, granular concentrates can be processed quickly and cleanly, while maintaining a high degree of accuracy. They are easy to store, which allows for the flexible coloring of relatively small amounts of products. The desired color effect depends on the type of polymer, the chosen processing method, and the requirements of the individual consumers.

An especially important role is played by the optimization of the dispersion of pigments and additives in the entire volume of the manufactured element. The colored concentrates consist of at least one or more pigments, which have been dispersed throughout the polymer support material. In the factory, each time a pigment formula is developed for a given concentrate color, it should be tailored to the expectations of the customer. Coloring agents often consist of various modifiers that provide the final products with guaranteed functional properties, e.g., resistance to temperature, UV radiation, etc. [14].

**Figure 4:**
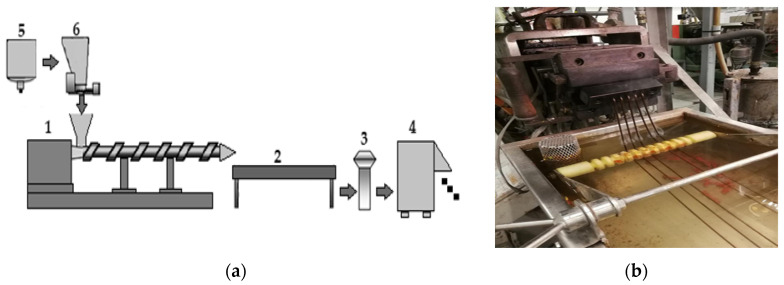
Elements of machines and devices constituting the technological line for the production (extrusion) process of coloring materials for polymers: (**a**) 1. Twin-screw extruder, 2. cooling bath, 3. dryer, 4. granulator, 5. polymer silo, 6. polymer dispenser; (**b**) view of the multi-hole head and cooling bath in the cold granulation process and granulator.

The basic device in the process of producing pigments is a technological line consisting of an extruder, whose task is to supply a plasticized material with an appropriate flow rate, temperature, pressure, and degree of material and thermal homogenization to the head [12,15]. In turn, the task of the extrusion head is to provide the processed material an appropriate shape, while ensuring its appropriate homogeneity, thereby taking into account the phenomenon of stream expansion (Barus effect). Another element in the technological line is to cool and fix the final shape of the extruded “thread” of the material [16]. Figure 4 shows the line for cold granulation of polymeric materials, including all machine and process elements.

### 2.3. Life Cycle Impact Assessment (LCIA)

The potential environmental impact of each life cycle process by a selected product is quantified in terms of health, ecosystem quality, and resource consumption.

As the main method for assessing the potential environmental impact of the manufacturing process (extrusion) of coloring materials for the selected life cycle assessment (LCA) of the plastic polymer. According to the ISO 14,000 standards, the LCA analysis should include four stages: determination of the goal and scope, life cycle inventory (LCI), life cycle impact assessment (LCIA), and interpretation [5,16].

The work evaluates the process of manufacturing (extrusion) coloring materials for polymers. The conducted research was aimed at a detailed, ecological, and energetic analysis of the life cycle of selected aspects in the production of coloring materials intended for polymer plastics. On the one hand, the studies performed were intended to describe the existing reality (retrospective LCA); however, attempts were also made to model future changes and define recommendations aimed at developing more environmentally friendly solutions (prospective LCA). The performed procedure was a classic LCA process, which was compliant with the guidelines contained in the ISO 14,000 standards. Here, the main task was to determine the extent of the negative impact of the life cycle of the analyzed facilities on the health of humans and animals, the environmental quality, and the depletion of natural resources.

The functional unit adopted for the research was determined according to the data collected from the manufacturing company. It specifies the production of one batch of coloring materials for polymeric materials. One batch of the finished product was defined as 100 kg of ready-made granules.

This means that all steps were recorded from the delivery of the pigments and polymers to the production plant until they were properly shaped in the process of producing the coloring materials for the polymers. Further stages in the production process, such as storing the finished product and transporting it to the customer have been excluded from the system.

### 2.4. ReCiPe 2016 Method

ReCiPe 2016 was developed in cooperation with the Dutch National Institute of Public Health and Environment (RIVM), Radboud University Nijmegen, the Norwegian University of Science and Technology and PRé. In this updated version, PRé and The Radboud University in Nijmegen have continued their cooperation over recent years in the development of mineral and fossil depletion.

The environmental assessment covered the seventeen impact categories that are available under the ReCiPe 2016 model: global warming, stratospheric ozone depletion, ionizing radiation, ozone formation, fine particulate matter formation, terrestrial acidification, freshwater eutrophication, marine eutrophication, terrestrial ecotoxicity, freshwater ecotoxicity, marine ecotoxicity, human carcinogenic toxicity, human non-carcinogenic toxicity, land use, mineral resource scarcity, fossil resource scarcity, and water consumption. The result of the analysis was the assessment of the impact in three areas:

− Human health: impacts of global warming, stratospheric ozone depletion, ionizing radiation, ozone formation, fine particulate matter formation, human carcinogenic toxicity, human non-carcinogenic toxicity, and water consumption. The unit of these interactions is DALY. The disability-adjusted DALY parameter was developed by Murray at Harvard University in collaboration with the World Health Organization (WHO) to quantify the burden of disease and injury on humans. It is a time-based measure, which combines the time lost due to premature death (indicating years of life lost, YLL) and the duration of disability caused by disease (years of life lived with a disability, YLD) in survivors. One DALY corresponds to one year of healthy life lost, which is equivalent to only 90% full capacity and life for 10 years.
− Impact on the quality of ecosystems: Impacts related to global warming, ozone formation, terrestrial acidification, freshwater eutrophication, marine eutrophication, terrestrial ecotoxicity, freshwater ecotoxicity, marine ecotoxicity, land use, and water consumption. The unit of quality of these impacts on ecosystems is the loss of local species integrated in time (year of survival of a given species) species per yr.
− USD 2013: Resource scarcity damage refers to the additional dollar cost (USD 2013) of future resource extraction. In this way, a wide variety of environmental effects are considered. As in the case of introducing economic factors, some environmental parameters depend on regional specificities, such as the country’s energy mix. These results can be found in some studies on environmental parameters. Four emission areas for individual chemical compounds were also specified, including air, water, soil, and raw [5,17,18,19].

Life cycle impact assessment was performed using the SimaPro 9.2.0.2. software (PRé Sustainability, LE Amersfoort, Netherlands) with the Ecoinvent 3.7.1 database. The cut-off level adopted for the research was 0.5%. The ecological analysis of the life cycle impact on the manufacturing (extrusion) process for polymer coloring materials was possible by using the ReCiPe 2016 method. The ReCiPe 2016 method belongs to the group of methods that model the environmental impact at the endpoints of the environmental mechanism. The characterization process occurred for eleven impact categories, which fell into three larger groups known as areas of influence. The following areas of influence were distinguished: human health, ecosystem quality, and resources. The results from the areas of influence indicators were subject to further aggregation in the final ecolabel through normalization, grouping, and weighting (Table 1) [14,20,21,22].

Normalization is understood as computing the magnitude of the results for a category indicator against the reference information. It is helpful in determining the relative importance of an indicator’s results relative to a particular region (e.g., Europe), or person (e.g., average European inhabitant) over a specific time period. Normalization can also be used to prepare LCIA results for subsequent procedures, e.g., weighting. The results of the indicator in the characterization stage are obtained in different units, meaning it would be difficult to assign specific weighting factors to them and then multiply them. However, conversion to a common unit through normalization allows for their subsequent weighting [14].

Weighting consists of determining and assigning a degree of importance (weighting factor) to individual impact categories before using them to multiply the normalized results of the index. Carrying out the weighting process allows the results to be obtained in environmental points (Pt), where a thousand environmental points are equal to the environmental impact of one European in one year [6].

## 3. Results

### 3.1. ReCiPe 2016 Method

The results of the analyzes carried out as part of the LCIA were summarized based on the used ReCiPe 2016 method. The modeling results with the ReCiPe 2016 method were divided into two groups: results in the field of the impact categories and areas of influence. A detailed analysis of the seventeen impact categories available under ReCiPe 2016 included global warming, stratospheric ozone depletion, ionizing radiation, ozone formation, fine particulate matter formation, terrestrial acidification, freshwater eutrophication, marine eutrophication, terrestrial ecotoxicity, freshwater ecotoxicity, marine ecotoxicity, human carcinogenic toxicity, human non-carcinogenic toxicity, land use, mineral resource scarcity, fossil resource scarcity, and water consumption.

The results for the manufacture (extrusion), coloring materials, and plastic polymer-supported PET stage of manufacture are summarized hereafter. The first step of the analysis included an assessment of the eighteen considered categories to determine which may be the source of potentially the greatest number of negative (or positive) environmental consequences in the life cycle (Table 4).

**Figure 5:**
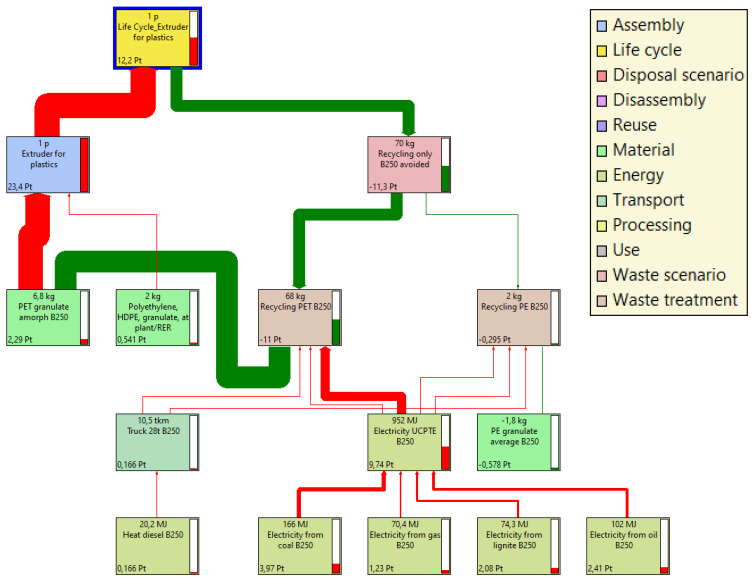
Process tree for the production of polymer coloring materials.

In the next stage, the inputs and outputs were analyzed for each case. As part of this step, a process tree was compiled, which showed the material flows throughout the analyzed life cycle. The level of impact of the presented components that were considered in the processes was determined at the 1% level. By limiting the level of impact of the presented components in the processes, one should understand the presentation of inputs and results for which the impacts are greater than the given percentage value of the impact. Therefore, in the analyzed example, the total negative impact was estimated at 23.4 Pt, although by using the recycling process the negative impact could be reduced by as much as −11.3 Pt/one batch of coloring materials for polymers (Figure 5).

### 3.2. Results of the Characterization

**Figure 6:**
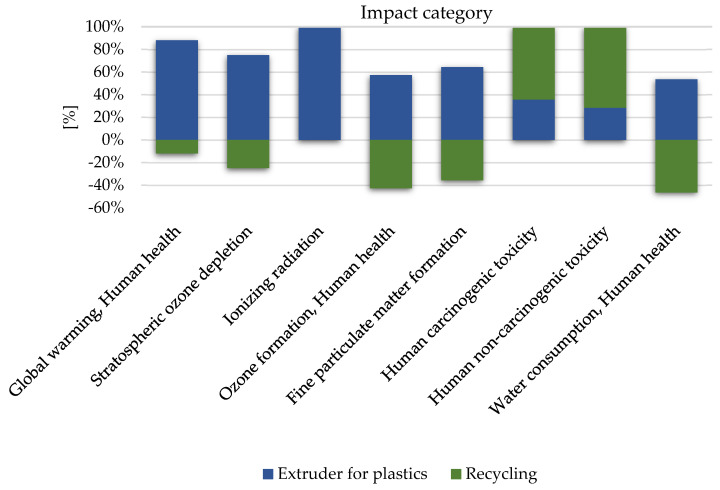
The results of the characterization of the environmental implications on the manufacturing process of coloring materials, plastic polymer-supported PET has an impact on human health.

Among the factors with a potential negative impact on human health (Figure 6), the group of fine particulate matter formation compounds, which had a value of 3.63 × 10^−4^ DALY, were characterized using the highest level of harmful effects. Recycling the studied plastics may significantly reduce their potential negative impact on the environment from the perspective of the production stage. The reduction value in this case is (−2.01 × 10^−4^ DALY). The key compound that shapes the size of the potential adverse impact on climate change in the life cycle of the production (extrusion) of polymer coloring materials was the emission of carbon dioxide into the atmosphere, where the total level of harmfulness was (−1.50 × 10^−4^ DALY).

**Figure 7:**
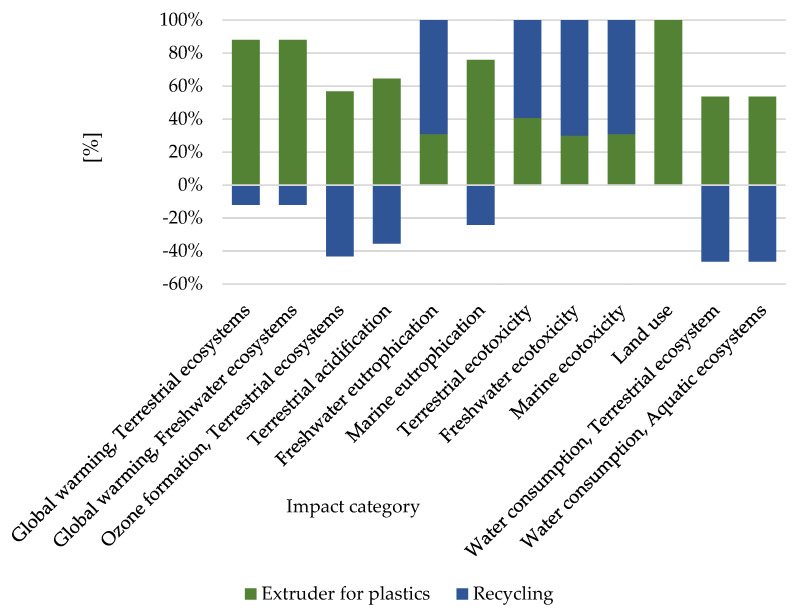
The results of the characterization of the environmental implications on the manufacturing process of coloring materials, plastic polymer-supported PET affect the quality of the external environment.

In the group of factors that contributes to the deterioration in the quality of the environment, 3 categories are highly important: global warming (4.53 × 10^−7^ species.yr), ozone formation (2.57 × 10^−7^ species.yr), and terrestrial acidification (4.17 × 10^−7^ species.yr). Analyzing the aspects related to the deterioration in the quality of the environment and, consequently, its impact on human health, the problem of the ozone hole enlargement is also important. The stratospheric ozone absorbs some of the ultraviolet radiation that reaches the Earth. Moreover, some types of radiation are harmful to living organisms because they can damage cells and the genetic material they contain. Acidification of the soil and surface waters occurs mainly by converting air pollutants into acids. The presence of acids in the environment leaches nutrients and increases the solubility of metals in the soil. This destroys ecosystems and leads to the acidification of surface waters, which destroys living organisms, mainly resulting from the dissolution of heavy metals. In turn, soil acidification causes disturbances in vegetation processes [23]. The reduction in the quality of the environment was designated the greatest value of 4.53 × 10^−7^ species.yr (Figure 7), and was caused by the emission of compounds. Recycling has the potential to reduce the harmful effects of global warming compounds to a level of −6.20 × 10^−8^ species.yr, which was the highest potential reduction in environmental damage.

**Figure 8:**
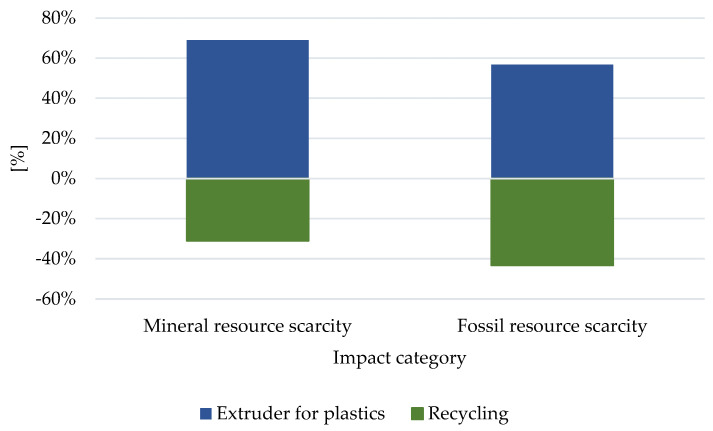
The results of the characterization of the environmental consequences on the manufacturing process of coloring materials for plastic, the PET polymer on the media affects the depletion of natural resources.

The main fossil fuels used in the production of polymers include inter alia, crude oil, coal, and natural gas [24,25]. The processes related to their extraction significantly reduce the quality of the environment [25,26]. In turn, among the factors related to the depletion of fossil resources (Figure 8), the extraction of mineral resources is by far the most detrimental (4.83 × 10^−4^ USD 2013). This is the result of high energy demand during the production of pigments for plastics and the related energy-consuming processes of extracting non-renewable raw materials. Recycling (2.20 × 10^−4^ USD 2013) can reduce the magnitude of the harmful effects in the area under consideration.

In order to identify the areas of the production (extrusion) process of coloring materials more precisely for polymer materials, which can potentially have the greatest negative (or positive) impact on the environment, a detailed analysis of substances and processes occurring within all the impact categories was performed (Table 5).

**Figure 9:**
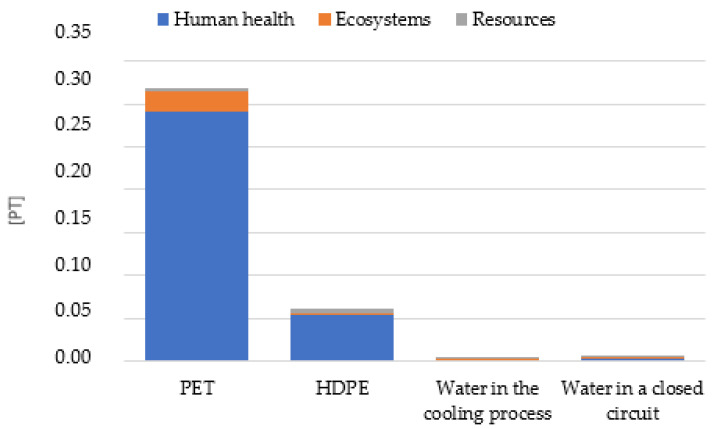
Characterization results of environmental consequences occurring at the stage of coloring materials production, categorized by materials and media used.

The process of producing coloring materials for polymers requires a lot of energy. It was noted that a particularly high level of potential negative impacts on human health for the two PET materials and the pigment in the HDPE carrier: ranging from 5.42 × 10^−2^ Pt for the pigment to 2.91 × 10^−1^ Pt for the PET (Table 5). Where water was used for the proper course in the production process, the highest potential emission level was recorded for water in a closed cycle (Figure 9), with a total of 2.11 × 10^−3^ Pt for ecosystems and 2.56 × 10^−3^ Pt for resources.

**Figure 10:**
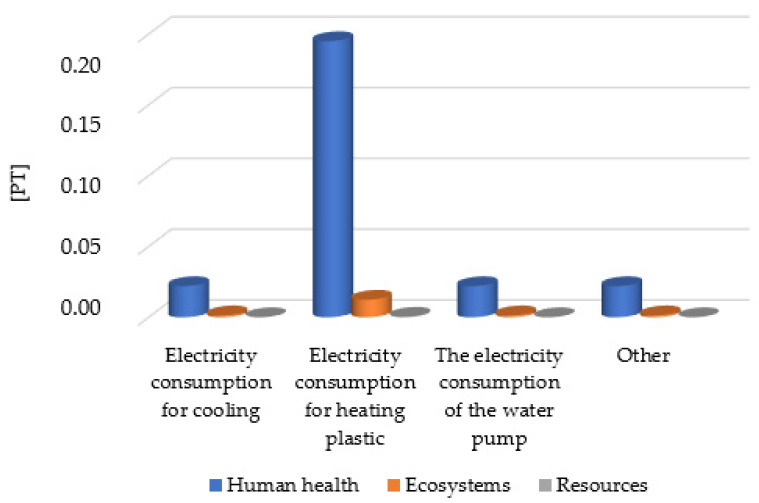
Characterization results of environmental consequences occurring at the stage of coloring materials production, categorized by electricity consumption.

The size of the total environmental impact is mainly the amount of electricity demand (Table 6). Although energy is currently obtained mainly from fossil fuels, it is more and more often recognized that this method of energy generation is the cause of environmental pollution and the depletion of natural resources [27,28,29,30]. The greatest potential level of negative environmental effects related to electricity consumption was the twin-screw extruder, which heats the plastic to the level of proper plasticization for the mixture of polyethylene terephthalate with the pigment. The second largest level of negative environmental impacts for ecotoxicity and fossil fuels was characterized by a cooling tank, where the tank is used to cool the finished product (Figure 10).

### 3.3. Results of Grouping and Weighing

**Figure 11:**
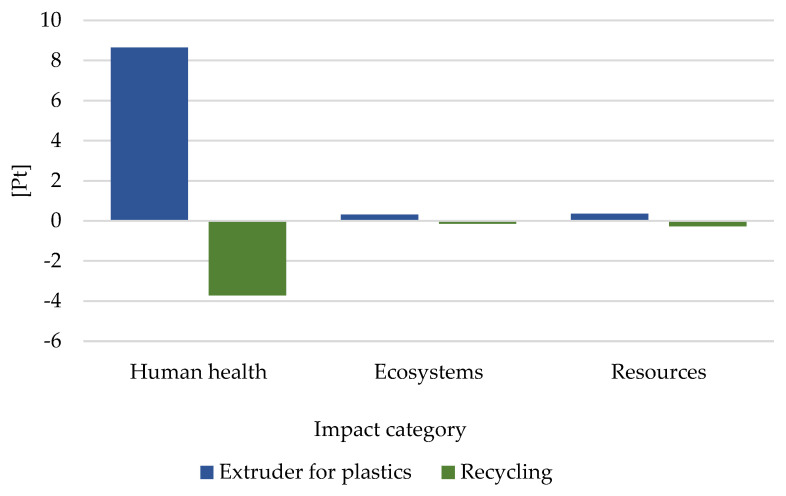
Grouping and weighing results of the manufacturing process of coloring materials, plastic polymer-supported PET.

Among the three assessed groups, the highest level of potential negative influence was recorded for human health in 9 Pt (Figure 11). The process of recycling all the considered wastes may cause (to a greater or lesser extent) a reduction in the level of negative impacts on human health, in relation to their entire life cycle.

## 4. Discussion

Models and adopted indicators were used to perform a state analysis on the basis of assumptions, while causal relationships for product quality, process efficiency, and harmlessness of the product were also assessed during the process of shaping sustainable development in a plastic dyeing environment. The following possibilities were indicated: to increase the quality of the product; increase in environmental, energy, and economic benefits; reduce the number of harmful gases, dust, waste, and emissions in plastic dyes, such as for cold granulation. The LCA strategy is an excellent tool for supporting the analysis, assessment, and, above all, integrated development in the technological processes of dyeing plastics.

Authors can determine the scope of the production plant’s impact on the natural environment by conducting a constant analysis of the production processes and operations as well as of the existing infrastructure. These constant analyses can be used to determine the environmental impact, indicate the possibilities of reducing or completely eliminating the impact, and assess the effects of the implementation of pro-ecological projects.

The tasks in the plastics processing plant included quantitative and qualitative analyses of processed raw materials as well as monitoring the energy consumption of carriers and the emission of pollutants into the atmosphere. One of the tools that can be used in this analysis, together with the identification of environmental problems, was the life cycle impact assessment. The compilation of the ecological balance made it possible to identify the above-mentioned areas in the production process, whereby material consumption may be reduced, energy efficiency increased, and the amount of pollutant emissions reduced. The manuscript shows the energy intensity in the production, material flows, and energy developed on the basis of data collected from the company in the plastics sector. The type of environmental impact depends mainly on the specificity of the industry and the chemical composition of the raw materials used. Production plants should, if possible, conduct an ongoing analysis of the technological processes, including the consumption of raw materials and energy carriers. In the presented study, the most important impact category was the amount of plastic polyethylene terephthalate being used (73% of the total environmental impact). By comparing the values for the environmental impacts being used in the media process, it was found that the potentially greatest environmental impact was associated with the use of water in a closed cycle (300 L).

This is because the analyzed process of producing pigments for plastics used in the cold granulation process requires adequate cooling of the raw material in a cooling bath (1.57 kWh). Moreover, the values for the environmental impact of electricity consumption were compared (in the entire process, its total level was 16.3 kWh). In addition, three main impact categories were identified throughout the study: The first, fine particulate matter formation (0.000162 DALY) resulted in a large number of plastic particles and powders being used in the production process. Next, the emission of greenhouse gases into the environment increased the greenhouse effect (0.00015 DALY). This harmful process is the result of materials based on fossil fuels being used for testing. Thus, replacing traditional plastics with materials derived from renewable resources can help to reduce greenhouse gas emissions and increase production efficiency and productivity. In addition, the four impact categories with the highest potential environmental impacts were noted throughout the assessment process: i.e., water consumption aquatic ecosystems (9.88 × 10^−14^ species.yr); global warming and freshwater ecosystems (1.07 × 10^−11^ species.yr), marine eutrophication (6.63 × 10^−13^ species.yr), and marine ecotoxicity (2.8 × 10^−11^ species.yr). Future research should be aimed at investigating and better understanding the potential environmental impacts of using feedstocks for plastic production.

## 5. Conclusions

The LCA results of the assessment process facilitate decision-making, which should improve the production processes for plastics processing. Additives should be included in the LCI for the LCA testing of plastics. Therefore, there is an urgent need for more studies that assess, in detail, the environmental impacts of additives, especially with regard to end-of-life processes. In connection with the above, there is a need to apply consequential LCA, to represent the environmental outcomes of widespread substitutions in petrochemical plastics with bioplastics.

## Figures and Tables

**Table 1:** Components of the material and energy balance from the perspective of environmental protection.

Inputs	Outputs
**MATERIALS**	**PRODUCTS**
− production raw materials − semi-finished products from other branches and industries − auxiliary raw materials and components − other materials and secondary raw materials	− basic products − by-products and production residues − semi-finished products for further processing − waste
**ENERGY**	**MATERIAL EMISSIONS**
− solid fuels − liquid fuels − gaseous fuels − water − steam generation − electricity − renewable energy − use of waste (from production) for energy purposes	− waste from the transformation of energy carriers and raw materials − sewage, production water, and sewage sludge − exhaust fumes − articulates and gaseous pollutants
	**ENERGY EMISSIONS**
	− heat losses from production equipment − noise

**Table 2:** Inventory table: consumption of energy carriers, raw materials, and materials in the production (extrusion) of coloring materials, using a PET-based coloring concentrate as an example.

Material	Amount
PET	76 kg/h
Pigment granules 0.4% in HDPE	28 kg
0.07% X + 1% TiO_2_ on HDPE support	
Cooling water quantity	1015 l
The amount of water in a closed circuit	1.29 l
Electricity consumption for the extrusion process	13.93 kWh
Electricity consumption for the cooling process	1.57 kWh
Electricity consumption for the operation of the water pump	0.5 kWh
Electricity consumption for other processes	0.3 kWh

**Table 3:** Production residues and waste.

Material	Amount
The amount of PET waste before starting the process	5 kg
Completion of the process, removal of raw material residues from the machine	3 kg
Water losses	300 L/week

Functional unit comprising 100 kg/1 p.

**Table 4:** The results of the characterization of the consequences of environmental problems in various stages of the life cycle for the manufacturing process (extrusion), coloring materials, and plastic polymer.

Impact Category	Unit	Total	Extruder for Plastics	Recycling
Global warming, human health	DALY	0.00013	0.00015	−2.1 × 10^−5^
Global warming, terrestrial ecosystems	species.yr	3.91 × 10^−7^	4.53 × 10^−7^	−6.2 × 10^−8^
Global warming, freshwater ecosystems	species.yr	1.07 × 10^−11^	1.24 × 10^−11^	−1.7 × 10^−12^
Stratospheric ozone depletion	DALY	2.54 × 10^−8^	3.81 × 10^−8^	−1.3 × 10^−8^
Ionizing radiation	DALY	5.75 × 10^−10^	5.75 × 10^−10^	0
Ozone formation, human health	DALY	4.01 × 10^−7^	1.58 × 10^−6^	−1.2 × 10^−6^
Fine particulate matter formation	DALY	0.000162	0.000363	−0.0002
Ozone formation, terrestrial ecosystems	species.yr	6.11 × 10^−8^	2.57 × 10^−7^	−2 × 10^−7^
Terrestrial acidification	species.yr	1.87 × 10^−7^	4.17 × 10^−7^	−2.3 × 10^−7^
Freshwater eutrophication	species.yr	7.11 × 10^−7^	2.19 × 10^−10^	4.92 × 10^−10^
Marine eutrophication	species.yr	6.63 × 10^−13^	9.73 × 10^−13^	−3.1 × 10^−13^
Terrestrial ecotoxicity	species.yr	4.9 × 10^−10^	1.99 × 10^−10^	2.91 × 10^−10^
Freshwater ecotoxicity	species.yr	1.25 × 10^−10^	3.73 × 10^−11^	8.77 × 10^−11^
Marine ecotoxicity	species.yr	2.8 × 10^−11^	8.62 × 10^−12^	1.93 × 10^−11^
Human carcinogenic toxicity	DALY	3.69 × 10^−7^	1.32 × 10^−7^	2.38 × 10^−7^
Human non-carcinogenic toxicity	DALY	3.41 × 10^−6^	9.7 × 10^−7^	2.44 × 10^−6^
Land use	species.yr	1.76 × 10^−10^	1.76 × 10^−10^	0
Mineral resource scarcity	USD2013	0.000265	0.000483	−0.00022
Fossil resource scarcity	USD2013	11.54808	48.9723	−37.4242
Water consumption, human health	DALY	3.63 × 10^−7^	2.74 × 10^−6^	−2.4 × 10^−6^
Water consumption, terrestrial ecosystem	species.yr	2.21 × 10^−9^	1.67 × 10^−8^	−1.4 × 10^−8^
Water consumption, aquatic ecosystems	species.yr	9.88 × 10^−14^	7.47 × 10^−13^	−6.5 × 10^−13^

**Table 5:** Characterization results of environmental consequences occurring at the stage of staining materials production, categorized by materials and media used.

Damage Category	Unit	PET	Pigment	Water in the Cooling Process	Water in a Closed Circuit
Human health	Pt	2.91 × 10^−1^	5.42 × 10^−2^	1.86 × 10^−3^	7.14 × 10^−3^
Ecosystems	Pt	2.35 × 10^−2^	2.18 × 10^−3^	1.42 × 10^−4^	2.47 × 10^−3^
Resources	Pt	4.23 × 10^−3^	4.56 × 10^−3^	2.49 × 10^−5^	2.11 × 10^−3^
Total	Pt	3.19 × 10^−1^	6.10 × 10^−2^	2.02 × 10^−3^	2.56 × 10^−3^

**Table 6:** Characterization results of environmental consequences occurring at the stage of coloring materials production, categorized by electricity consumption.

Damage Category	Unit	Electricity Consumption for Cooling	Electricity Consumption for Heating Plastic	The Electricity Consumption of the Water Pump	Other
Human health	Pt	2.20 × 10^−2^	1.95 × 10^−1^	2.20 × 10^−2^	2.20 × 10^−2^
Ecosystems	Pt	1.40 × 10^−3^	1.24 × 10^−2^	1.40 × 10^−3^	1.40 × 10^−3^
Resources	Pt	4.13 × 10^−5^	3.66 × 10^−4^	4.13 × 10^−5^	4.13 × 10^−5^
Total	Pt	2.34 × 10^−2^	2.08 × 10^−1^	2.34 × 10^−2^	2.34 × 10^−2^

## Data Availability

The data presented in this study are available on request from the corresponding author.
